# European paramedic curriculum—a call for unity in paramedic education on a European level

**DOI:** 10.1186/s13049-021-00889-z

**Published:** 2021-05-31

**Authors:** Sveinbjörn Dúason, Christoffer Ericsson, Hrafnhildur Lilja Jónsdóttir, Jeanette Viggen Andersen, Thomas Lynge Andersen

**Affiliations:** 1 School of Health Sciences, University of Akureyri, Norðurslóð 2, 600 Akureyri, Iceland; 2 Department of Healthcare, Arcada University of Applied Sciences, 00560 Helsinki, Finland; 3 Oslo Metropolitan University, Pilestredet 32, 0167 Oslo, Norway; 4 EMS Copenhagen, Capital Region, Telegrafvej 5, opg. 2, 2750 Ballerup, Denmark

**Keywords:** Ambulance transport, Curriculum, Emergency Medical Service, Paramedic BS degree, Paramedic education, Prehospital

## Abstract

**Background:**

There have been major developments in healthcare services as well as changes in demographics in recent years, and this has, among other things, led to increased demand for ambulance services. In general, this has also led to calls for more highly educated paramedics.

**Main body:**

Erasmus + provided a grant for three universities and one public service provider in four Nordic countries to work on a harmonised model curriculum for a bachelor’s degree in paramedic education. The project group has now completed the first phase of the project, which was to examine what paramedic education is available in the participating countries and what laws and regulations affect both the operation of ambulance services and the education of paramedics. At the end of the project, a harmonised exemplary curriculum will be available to anyone interested in educating paramedics at the university level.

**Conclusions:**

The growing need for highly educated paramedics should be addressed by offering a bachelor’s degree university education with an exemplary curriculum and coordinating it within Europe. The added value of a harmonised education programme within Europe would thus enable further and deeper collaboration.

## Background

The evolution and centralisation of healthcare service units and significant demographic changes put pressure on the delivery of healthcare services, and therefore, on ambulance service technicians [1], who, regardless of education level, are hereafter referred to as paramedics. This shift has changed the prehospital service from the traditional role of focusing on first aid, stabilisation and rapid transportation to conducting thorough and systematic patient assessments, recognising acute conditions and initiating advanced treatment, often in emergency and time-critical situations. Paramedics further offer community health services, aid in acute mental health and social distress issues, and more often provide treatment that was previously only available within hospitals. This responsibility demands a higher level of competencies among paramedics. Cross-border co-operation, in, among others, major accidents and mass casualty incidents, also requires paramedics to have comparable knowledge bases and be able to provide equivalent services between countries.

As a result of the increased responsibility and role change, higher-level education has been called for in paramedicine (http://www.stjornarradid.is/media/velferdarraduneyti-media/media/ritogskyrslur2011/IIceland_HCS-Final_report-_Long_version.pdf) Some countries are aiming to educate and graduate paramedics at the university level with a bachelor’s degree (BS degree). However, the education and training of paramedics varies within European countries [2, 3], even within neighbouring countries, such as the Nordic countries [4, 5]. Some require a BS degree, while others have shorter coursework at a lower academic level. Finland has more than 20 years of experience in educating paramedics at the university level, initiating its Emergency Care Programme in 1998. Norway founded a Paramedic BS degree programme in 2014, while Denmark and Iceland are in the preparation stage for teaching paramedicine at the university level. However, to our knowledge, there has not yet been an attempt at structuring a harmonised curriculum that could reach across international borders. As previously noted, it would seem desirable that paramedics have a similar academic background and qualifications. We need to respond to this reality. As a start, three universities and one public service provider from four Nordic countries have decided to work together with the objective of creating an ‘exemplary curriculum’ for the education of paramedics at the university level [6] (Bachelor of Science, minimum 180 ECTS, according to Bologna principles). The target groups are universities and health authorities in Europe that are considering offering this programme.

A harmonised exemplary curriculum can be beneficial for both universities that are in the preparatory phase and also for those who are already teaching paramedicine as a BS degree. This might enable the latter to receive an ‘exemplary curriculum’ that could be used to compare to their existing curricula. Educational authorities could use it either in its full form or as a reference for evaluating and proposing amendments and developments in their existing educational content. A harmonised exemplary curriculum could potentially increase the likelihood that more European countries would offer a paramedic education programme at the university level. Consequently, this would improve the quality of service in paramedic health care.

The aim of the project is to produce a uniform harmonised curriculum-based framework for paramedic education at the bachelor’s degree-level, with potential for adaptability across European countries. The goal is to create an attractive educational training programme for students seeking a higher level of paramedicine education in countries that do not offer this, raising the potential for more motivated paramedic practitioners and even longer and wider career options for the future.

## Main text

Three universities and one public service provider in four Nordic countries received a grant from Erasmus + to work on an exemplary curriculum for a university bachelor degree education for paramedics. In addition to the applicant (the University of Akureyri Iceland who will coordinate the project), these institutes include the Arcada University of Applied Sciences in Finland, Oslo Metropolitan University in Norway and the Capital Region Emergency Medical Service in Denmark. It is a strength of the project that the paramedic education in these countries is different; hence, the best practices from each of these different collaborators can be exploited to create a harmonised exemplary curriculum for paramedic education.

This project is divided into two phases; the first part is presented here. The results of the first part will be used as the groundwork for the second section.

In the first part, we have gathered information on the status of paramedic education from all participating countries and any laws and regulations affecting the work and education of paramedics. The aim was to compare differences and explore to what degree different laws in different countries potentially affect curricula.

In the second part, we will attempt to compare the prerequisites for studying university-level paramedic degrees and compile and compare existing curricula for paramedic studies in, at minimum, the participating countries. We will try to predict how paramedic work will evolve in the future, for example, related to the use of artificial intelligence, mobile connections and applications, and how universities and curricula could follow or stay ahead of that development. We are aware that these will be, at most, educated guesses and speculations. We will also propose ways to effectively connect learning goals and relevant competencies to the use of simulation, as it can be challenging to bring students to specialised vocational training. We assess the value of adding ‘Community Paramedicine’ content to the curriculum, as it calls for paramedics to increase their responsibilities by providing on-site services in patients’ homes instead of transporting them by ambulance to a hospital. The final work, the exemplary curriculum, will be available for free as open-source material, the emphasis being that this curriculum is optional, and there is no obligation for any European nation to use or implement it.

## Paramedic education in the Nordic countries

There are differences in the education of paramedics among the participating countries. Various reasons for this have been noted, including historical ones. One reason is that both the education and practice of prehospital patient transport have developed differently in different countries. This paper will not detail the various forms of managerial operations of ambulances (this will be the subject of another manuscript); rather, the focus here is on the educational aspects of paramedics.

In Finland, there are two levels of paramedic work: advanced level (equivalent to advanced paramedic) and basic level (equivalent to emergency medical technician [EMT]). Working in prehospital care, at either the basic or advanced level, primarily requires a healthcare degree and registration by the Finnish National Supervisory Authority for Welfare and Health (Valvira). An EMT qualification diploma—applicable for emergency care students after the EMT course and also professionally trained firefighters—also suffices for the basic level. The educational requirements for basic-level competency are a practical nurse with prehospital specialisation, basic-level paramedic or registered nurse. Advanced level competency is either a registered nursing degree with completion of a 30 ECTS advanced-level prehospital specialisation course or a bachelor-level emergency care degree.

In Norway, there are two levels of prehospital education: advanced level (equivalent to paramedic) and basic level, which is called ambulance service technician (equivalent to EMT) in Norway. The basic level degree is primarily obtained with a combination of secondary education and clinical practice, while the paramedic BS degree is attained at the university level. Nevertheless, there are multiple internal courses within the different ambulance services to obtain an advanced EMT level. There are also personnel without formal prehospital education working in the ambulance service. By law, every ambulance is obligated to have at least one authorised EMT personnel at all times.

In Denmark, there are two prehospital educational levels: EMT and paramedic. EMT education is situated within the Ministry of Transport due to the history of ambulance workers with no healthcare education. The education lasts for four and a half years, but the first year is combined with other education at a basic vocational training level in working life and is associated nationally with two vocational schools (secondary level) by bid. The current paramedic education programme in Denmark is associated with a university college, but the curriculum has no valid healthcare degree as a target. However, the paramedic education provides access to national registration as a paramedic. Paramedic education in Denmark has existed for over 15 years but has not developed into a formal educational system as a degree or equivalent.

In Iceland, the EMT (Paramedic) School offers programmes for EMTs on two levels based on the United States National Standard Curriculum: EMT 260 h and Advanced EMT 350 h. In addition to the EMT course, there are also a few other shorter courses. After graduation as an EMT, one can apply for a licence to practice from the Directorate of Health. To qualify as a paramedic, students must go abroad, for example, to the United States. The programme there takes 9 to 12 months, depending on where it is taught. There is no paramedic education in Iceland at the university level.

## Laws affecting the work and education of paramedics

In the aforementioned countries, ambulances, patient transport, operations and paramedic education have developed in line with the legal frameworks and working environments of the profession. This means that to be able to formulate a harmonised curriculum, it is necessary to compare both the legal frameworks and the work processes/guidelines that paramedics follow. Therefore, part of our work was to review and compare laws and regulations related to the subject. The legal issue questions we had for reference were the following:

- Which laws and regulations relate to ambulance services and/or out-of-hospital services?
- What are paramedics allowed to do regarding medication administration and independent decision-making?
- Who is the responsible party and for what? Who has medical responsibility?
- In what roles are paramedics allowed to work exclusively in ambulances or emergency rooms and in hospital? Does this overlap somewhere?
- What is the relationship with the civil protection system and other professions?
- Has a need for legislative changes been relevant? Considering consistency between participating countries would be desirable.
- Are paramedics registered as healthcare professionals?

All participating countries have largely similar legislation regarding the healthcare system. There are laws on patients’ rights, the Act on the Rights and Duties of Healthcare Professionals as well as several other related laws. There is a difference in the general education of paramedics, and they work according to different work processes. These dictate certain rules, such as medication administration rights and levels of autonomy. Therefore, it may be necessary to change work processes in some countries if ambulance crews are to operate in a harmonised manner. We are not proposing such a massive change here but rather point this out, since prehospital emergency services provided to patients are comparable, regardless of the forms of operation and legal environments. Thus, it should be possible and sensible to have a similar curriculum, not merely for the participating countries but also on a European level.

## Conclusions

There is a growing need for highly educated paramedics within European countries. One factor in response to this is to provide such education at the university level. Nationally, there are different forms of managerial operations, traditions in ambulance patient transport and paramedic education that need to be considered. However, we believe that a European Paramedic Curriculum (EPaCur) could potentially increase the number of nations offering such an education at the bachelor’s degree level, thus raising the level of competency of paramedic practitioners overall.

## Data Availability

Not applicable.

## Abbreviation

EMT: Emergency Medical Technicians
